# Ethernet Passive Mutual Authentication Scheme on Quantum Networks

**DOI:** 10.3390/e27020135

**Published:** 2025-01-27

**Authors:** Jianuo Tian, Panke Qin, Zongqu Zhao, Baodong Qin

**Affiliations:** 1 Jiaozuo Technician College, Jiaozuo 454000, China; 2 School of Software, Henan Polytechnic University, Jiaozuo 454000, China; 3 Shaanxi Key Laboratory of Information Communication Network and Security, Xi’an University of Posts & Telecommunications, Xi’an 710121, China

**Keywords:** ethernet passive optical network (EPON), ideal lattices, mutual authentication, ring learning with errors (RLWE), approximate smooth projection hash function, security performance evaluation, quantum networks, quantum security

## Abstract

In the context of increasing demand for secure and efficient communication networks, addressing the issue of mutual authentication in ethernet passive optical networks (EPONs) has become both valuable and practically significant. This paper proposes a solution based on ideal lattices. The proposed scheme leverages the security of the ring learning with errors (RLWE) problem to establish a robust public-key cryptosystem. By involving ONUs, OLTs, and an SDN controller in the authentication process, it enables mutual authentication through a series of message exchanges facilitated by the SDN controller. Utilizing approximate smooth projection hash functions for secure key exchange and verification, the scheme ensures robust security performance against various attacks, including man-in-the-middle, impersonation, replay, and known key secrecy attacks. Simulation results demonstrate that the proposed solution introduces minimal delay and maintains a high registration success rate compared to traditional authentication methods. Additionally, this paper explores the convergence of quantum network protocols with EPONs, highlighting their potential to achieve unprecedented levels of communication security. Integrating quantum technology with EPON networks, due to the unique security properties of quantum, can also better prevent man-in-the-middle attacks. Secure interception detection techniques based on fundamental quantum properties provide a fundamental security direction for future communication systems, aligning with the growing interest in quantum-resistant cryptographic protocols.

## 1. Introduction

According to the 49th Statistical Report on Internet Development in China [1], the number of Internet users in China has reached 1.032 billion, marking an increase of 42.96 million compared to December 2020. Concurrently, the Internet penetration rate has risen to 73.0% by December 2021. In addition, the number of broadband access ports has escalated to 1.018 billion, and the cumulative length of optical cable lines has extended to 54.88 million kilometers. The advent of software-defined networking (SDN) represents a novel network architecture capable of defining and controlling networks through software programming, thereby enhancing the flexibility of next-generation networks, particularly within the 5G domain [2,3]. With fiber-to-the-home (FTTH) or FTTx as the vanguard of optical fiber deployment, the coverage of ethernet passive optical networks (EPONs) has garnered widespread public attention and acclaim as a critical step in fiber-to-home initiatives [4,5,6]. However, the unique point-to-multipoint topology of EPONs, where downstream information is broadcast to each ONU via TDM, presents inherent security risks. ONUs receive packets destined for them through LLID filtering rules, a process that, while simple, is fraught with insecurity. Malicious users can intercept all downstream data by disabling these filtering rules or, worse, forge legal LLIDs to infiltrate the system and launch attacks. Consequently, the EPON network is inevitably exposed to a myriad of security threats. In today’s increasingly information-centric society, safeguarding user privacy within the network has become a universally pertinent issue.

Although IEEE 802.3ah has established regulations for the automatic discovery and ranging of ONUs in EPON systems, it lacks the inclusion of authentication protocols [7]. To address this deficiency, Roh [8] proposed the establishment of public key exchange-based authentication and key agreement at the RS layer, situated between the physical and MAC layers. Roh [9] further designed a security protocol for the EPON MAC layer, based on a modified ECC emulation of the Diffie–Hellman mechanism for session key exchange protocols, aiming to reduce the overhead of security services. Focusing on the 10G EPON system, a security mechanism [10] based on GCM has been proposed, featuring a new mechanism for updating and synchronizing keys to ensure their accuracy and synchronization. In the authentication aspect, a detailed design process for ONU and user authentication using the GMAC module has been crafted, enabling the generation of an authentication mark attached to the data frame load. Inácio [11] suggested encrypting the preamble of data units to ensure frame-to-frame uniqueness, thereby enhancing system security, albeit at the cost of increased system delay. Zhu [12] proposed a scheme that integrates two-way authentication and hash functions, with security predicated on the Diffie–Hellman hypothesis and a target anti-collision hash function. Subsequently, Yin [13] introduced an integrated security scheme encompassing the number theory research unit signature algorithm (NTRUSign) for authentication and the AES encryption algorithm for data encryption, complemented by the elliptic curve Diffie–Hellman algorithm for key exchange in subsequent data transfers.

To counter degradation attacks in next-generation passive optical networks, Atan [14] proposed a detection and mitigation scheme. By constructing a collision detector, this scheme not only detects the ONU transmissions and their parameters but also monitors the number of conflicts within the network for each ONU. Subsequently, bandwidth penalties are imposed on detected malicious ONUs, while legitimate ONUs are permitted additional bandwidth allocation in subsequent cycles. To tackle the potential for man-in-the-middle attacks in EPON, Tsompanoglou [15] conducted a formal analysis to quantitatively assess the impact of illegitimate OLT attacks on EPON mechanisms and proposed a scheme to mitigate this impact. Jesus Martinez-Mateo [16] introduced a scheme for incorporating quantum cryptography into optical access networks, detailing the seamless integration of a quantum key distribution system.

To ensure the security of both ONUs and OLTs, we have adopted a more efficient protocol based on authentication elements [17], designed to generate sensitive information necessary for authentication, such as validation elements, random values, and temporary keys. Notably, among the frames transmitted for ONU registration, five are defined by the MPCP protocol of the EPON system, while the remaining three—ONU_CERTIFICATION, SDN_CERTIFICATION, and OLT_CERTIFICATION—are authentication frames we have designed. Furthermore, we introduce an SDN controller as a third party to assist in identity authentication and negotiate a session key for subsequent data encryption. Most importantly, the session key maintains its privacy from the SDN controller, thereby enhancing the overall security of the system.

Furthermore, the integration of secure quantum network communication protocols into traditional communication frameworks such as EPON is an emerging area of research. These networks exploit the unique physical properties of quantum to ensure secure key distribution, which is a critical component in establishing secure communication channels. 

The advent of quantum networks represents a significant leap forward in the realm of secure communication. Quantum networks exploit the principles of quantum mechanics to ensure secure key distribution, which is a critical component in establishing secure communication channels. The successful implementation of quantum key distribution (QKD) systems within optical access networks, as suggested by Martinez-Mateo [16], marks a significant step towards realizing the full potential of quantum-secure communication infrastructures. Recent advancements in photonic quantum network transmission, aided by weak cross-Kerr nonlinearity, have been reported by Wang et al. [18], showcasing the potential for enhanced quantum communication protocols. Furthermore, Luo [19] has contributed to the field by developing computationally efficient nonlinear Bell inequalities for quantum networks, enhancing our ability to test and verify the integrity of quantum communication systems.

The concept of nonsignaling causal hierarchy in multisource networks has been explored by Luo [20], providing a deeper understanding of the causal relationships within quantum networks. Luo [21] has also proposed a fully device-independent model for quantum networks, which is a significant advancement in ensuring the security and reliability of quantum communication without relying on the trustworthiness of the devices used. Jiang et al. [22] have delved into the network capacity of entangled quantum Internet, offering insights into the scalability and efficiency of quantum networks.

The vision of a quantum internet has been eloquently outlined by Kimble [23], who discusses the potential for a new era of communication based on quantum entanglement. Castelvecchi [24] provides a balanced view on the current state of the quantum internet, highlighting both the progress made and the challenges that remain. Finally, Azuma et al. [25] present a comprehensive review on quantum repeaters, which are essential for extending the range of quantum networks and realizing the quantum internet.

Although the IEEE 802.3ah standard proposes automatic discovery and ranging mechanisms for the physical layer and Media Access Control (MAC) layer of EPON systems, it does not include specific authentication protocols [8]. Traditional authentication schemes are typically based on ECC or symmetric encryption algorithms, employing cryptographic techniques for authentication and key exchange [10]. However, these schemes [8,9,10,11,12,13,14,15,26,27] exhibit significant shortcomings when faced with the threats posed by quantum computing. In recent years, the development of quantum computing has rendered traditional cryptographic systems based on integer factorization and discrete logarithm problems highly vulnerable, making it urgent to explore novel solutions in the field of post-quantum cryptography.

Ideal lattices, as a post-quantum cryptographic technology, offer strong resistance to quantum attacks due to the hardness of the Ring Learning with Errors (RLWE) problem. The security of RLWE is not only robust against quantum adversaries but also provides efficient implementations. This combination of high security and efficiency makes RLWE an attractive choice for practical cryptographic applications. Combined with Quantum Key Distribution (QKD) technology, they can further enhance the security of network communications, achieving dual protection at both the physical and cryptographic layers [16]. Additionally, the introduction of SDN brings new flexibility and control capabilities to the EPON authentication framework [1].

Our contributions

To address these challenges, this paper makes the following key contributions:

(1) First, we proposed a robust mutual authentication scheme for EPONs based on the RLWE problem, establishing a resilient public key cryptosystem capable of resisting quantum computing attacks and incorporating QKD technology to enhance communication security.
(2) Second, by incorporating the approximate smooth projection hash function, the proposed scheme enables secure key exchange and mutual authentication between ONUs and OLTs with the assistance of an SDN controller.
(3) Third, the proposed scheme guarantees strong security performance against a range of attacks, including man-in-the-middle, impersonation, replay, and known key secrecy attacks, highlighting its robustness and effectiveness.
(4) Finally, comprehensive simulation results demonstrate that the proposed scheme introduces minimal delay and maintains a high registration success rate, even under high load conditions.

The remaining sections of this paper are organized as follows: Section 2 covers some preliminaries. Section 3 provides a detailed introduction to our proposed scheme. Section 4 compares our strategy with others in terms of security and efficiency. Section 5 concludes the entire paper.

## 2. Preliminaries

### 2.1. Ideal Lattice

The set of points consisting of n linearly independent vectors $v_{1},\cdots v_{n}$ on the linear space $R_{n}$ is known as lattice, which can be expressed as $L=\{a_{1}v_{1}+\cdots +a_{n}v_{n}|a_{i}\;\mathrm{is}\;\mathrm{an}\;\mathrm{integer}\}$, where $v_{1},\cdots v_{n}$ is called the basis of this lattice. An ideal lattice [28] is a lattice with a special ring structure. Ideal Γ is a quotient ring of the ring R=ℤ[x]/f(x), where f(x) is the first integer polynomial. The set of Γ on the integer group ${\mathbb{Z}}^{n}$ of degree n is the ideal lattice.

### 2.2. Public-Key Cryptosystem Based on Ideal Lattice

The public-key cryptosystem is predicated on the ring learning with errors (RLWE) problem [28,29], which is fundamentally composed of three key algorithms:

(1) **Key generation algorithm:** Taking the security parameter n as input, and the public key PK and the private key SK are output, which is recorded as $\left(PK,SK)\leftarrow KeyGen(1^{n}\right)$.
(2) **Encryption algorithm:** Taking public key PK and plaintext m as inputs, and output the ciphertext C, which is marked as C←Enc(PK,m).
(3) **Decryption algorithm:** It takes the private key SK and ciphertext C as inputs, and output the plaintext m or rejection symbol ⊥, and record it as m←Dec(SK,C).

The ring learning with errors (RLWE) problem is a post-quantum cryptographic primitive that provides security against both classical and quantum attacks. It is based on the computational hardness of certain lattice problems, which involve solving noisy linear equations in high-dimensional spaces. This property makes RLWE a popular choice for encryption and authentication protocols in quantum-resistant cryptography.

### 2.3. Approximate Smooth Projection Hash Function

The concept of the smooth projection hash function originates from the pioneering work of Cramer [30] in the field of cryptography. Subsequent enhancements to this function have been instrumental in the development of efficient Password-Authenticated Key Exchange (PAKE) protocols, as notably contributed to by Katz [31] and Benhamouda [32].

$C_{pk}$ signifies the effective ciphertext space produced by public key pk encryption, and P denotes the plaintext space. We define set X and language L⊂X as follows:

$$X=\{(c,m)|c\in C_{pk};m\in P\},\;{\bar{L}}_{m}=\{(c,m)\in X|c=Enc_{pk}(m,r),r\in {\left\{0,1\right\}}^{*}\},\bar{L}=U_{m\in p}{\bar{L}}_{m},\;L_{m}=\{(c,m)\in X|m=Dec_{sk}(c)\},L=U_{m\in p}L_{m}.$$

In a nutshell, it is a double-key hash function. Given the X and L⊂X, the corresponding hash value of any word c∈L can be calculated in two ways. One is adopting the hash key hk and c, and the other is employing projection key hp and evidence w corresponding to c∈L. Specifically, the smooth projection hash function consists of four algorithms, which can be written as APSH=(HashKG,ProjKG,Hash,ProjH).

(1) $hk\leftarrow HashKG(1^{n})$: Enter the security parameter n, and the hash key generation algorithm outputs the hash key hk.
(2) hp←ProjKG(hk,pk): Given the hash key hk and public key pk, it will output the homologous projection key hp.
(3) h←Hash(hk,L,c): Let hash key hk, language L and any word $c\in \bar{L}$ be the input, and it yields hash value h.
(4) $h^{\prime }\leftarrow \mathrm{Pr}ojH(hp,w)$: When it inputs projection key hp and evidence w of any word $c\in \bar{L}$, the projection function outputs the projection key $h^{\prime }$.

**Correctness:** $h=h^{\prime }$ holds for any words c∈L and its corresponding evidence w.

**Smoothness:** For ∀c∉L, even if hp is known, Hash(hk,L,c) is statistically indistinguishable from completely randomly selected output.

### 2.4. Approximate Smooth Projection Hash Function on Ideal Lattice

(1) **Hash key:** The hash key space $HK={\left(R_{q}^{m}\right)}^{n}$ is designed to assure the approximate correctness of ε−ASPH function. For any $(e_{1},e_{2},\cdots ,e_{n})\in HK$, whose coefficients have to abide Gaussian distribution $\chi_{\beta }$.
(2) **Projection key:** $HP={\left({\mathbb{Z}}_{q}^{n}\right)}^{n}$ is the projection key space and the corresponding projection key $(u_{1},u_{2},\cdots ,u_{n})=\alpha (e_{1},e_{2},\cdots ,e_{n})\in HP$ for any $(e_{1},e_{2},\cdots ,e_{n})\in HK$. The detailed computation procedure is as follows: $u_{j}={\left(Map_{M-v}(e_{j})\right)}^{T}\cdot {\hat{B}}_{0}(j\leq n)$, where $Map_{M-v}(e_{j})$ is consequence of attaching the coefficients of polynomial of $e_{j}\in R_{m}^{q}$, the ultimate output is a one-dimensional column vector consisting of coefficients $e_{j}$. They will dot product ${\hat{B}}_{0}$ after all the coefficients $e_{j}\in (e_{1},e_{2},\cdots ,e_{3})$ have performed the last step. ${\hat{B}}_{0}$ is generated from public parameter B0←Rr through the following manipulation,
  $$B_{0}=\left[\begin{array}{l} b_{01}\\ b_{02}\\ \cdots \\ b_{0m} \end{array}\right],\;{\hat{B}}_{0}=\left[\begin{array}{l} rot{\left(b_{01}\right)}^{T}\\ rot{\left(b_{02}\right)}^{T}\\ \cdots \\ rot{\left(b_{0m}\right)}^{T} \end{array}\right]\in {\mathbb{Z}}^{mn\times n}.$$
(3) **Hash function** ${\left(H_{hk}\right)}_{hk\in HK}$: Enter the hash key $hk=(e_{1},e_{2},\cdots ,e_{n})\in HK$ and x=(c,m), the following calculation is performed $z_{j}={\left(Map_{M-v}(e_{j})\right)}^{T}\cdot (Map_{M-v}(c_{2}-B_{2}\cdot m))\in {\mathbb{Z}}_{q}$, where $m\in {\mathbb{Z}}_{q}^{n}$ and the output is
  $$b_{j}^{\prime }=\left\{\begin{array}{l} 0,if\;z_{j}^{\prime }<\frac{\left(q-1\right)}{2}\\ 1,if\;z_{j}^{\prime }>\frac{\left(q-1\right)}{2} \end{array}\right..$$
(4) **Projection function** ${\left\{\mathrm{Pr}ojH_{hp}\right\}}_{hp\in HP}$: The projection key is $hp=(u_{1},u_{2},\cdots ,u_{n})$ and the evidence w of $x\in \bar{L}$, then carry out the following computation: $z_{j}^{\prime }=u_{j}w\in {\mathbb{Z}}_{q}$. Obtain the output
  $$b_{j}^{\prime }=\left\{\begin{array}{l} 0,if\;z_{j}^{\prime }<\frac{\left(q-1\right)}{2}\\ 1,if\;z_{j}^{\prime }>\frac{\left(q-1\right)}{2} \end{array}\right..$$

The approximate smooth projection hash (ASPH) function is a specialized hash function used in lattice-based cryptography. ASPH maps high-dimensional lattice points into a smaller space while maintaining the statistical properties required for secure authentication. In this protocol, ASPH ensures that authentication messages are both efficient and secure against quantum adversaries.

### 2.5. Quantum Key Distribution Technology

Quantum key distribution (QKD) leverages the fundamental principles of quantum mechanics to enable secure key sharing between communication parties. The classical BB84 protocol, based on single-photon states, uses random basis selection and the measurement disturbance principle to achieve secure key distribution. Its core steps and formulas are as follows:

(1) **Quantum State Preparation:** The sender randomly selects a bit value $b_{i}\in \{0,1\}$ and a basis $\theta_{i}\in \{+,\times \}$, then prepares the corresponding quantum state {|0〉, |1〉, |+〉, |−〉}. These quantum states are transmitted to the receiver via a quantum channel.
(2) **Quantum State Measurement:** The receiver randomly selects a measurement basis $\theta_{i}^{\prime }\in \{+,\times \}$ and measures the received quantum state, obtaining the result $b_{i}^{\prime }$. The probability of the receiver’s measurement outcome depends on whether Alice’s and receiver’s bases match: $P(b_{i}^{\prime }=b_{i})=\left\{\begin{array}{cc} 1 & \;\mathrm{if}\;\theta_{i}=\theta_{i}^{\prime }\\ \;1/2 & \;\mathrm{if}\;\theta_{i}\neq \theta_{i}^{\prime } \end{array}.\right.$ If the bases match, the measurement result will always be consistent with the sent bit value; otherwise, the result is random.
(3) **Basis Reconciliation and Key Extraction:** Using a classical channel, the sender and receiver publicly disclose their chosen bases $\left(\theta_{i},\theta_{i}^{\prime }\right)$ and retain only the bits where the bases match $\left(\theta_{i}=\theta_{i}^{\prime }\right)$, forming the raw key: $K_{\mathrm{raw}}=\{b_{i}:\theta_{i}=\theta_{i}^{\prime }\}.$
(4) **Eavesdropping Detection:** By comparing a subset of the bits, the sender and receiver estimate the quantum bit error rate (QBER). If the QBER exceeds a predefined security threshold, it indicates potential eavesdropping on the quantum channel, and the communication is aborted.
(5) **Key Post-Processing:** Through error correction and privacy amplification, the sender and receiver transform the raw key $K_{\mathrm{raw}}$ into a final secure key $K_{final}$.

BB84 protocol can be directly applied to describe the key negotiation process between the SDN controller and ONU/OLT, providing a robust foundation for mutual authentication and data encryption in ethernet passive optical network schemes [16].

## 3. Proposed Scheme

This paper introduces an authentication scheme that facilitates a three-party password-based authentication key exchange on the ideal lattice. The scheme is designed to achieve mutual authentication between the optical network unit (ONU) and the optical line terminal (OLT) during the automatic registration process of the ONU. Concurrently, both parties negotiate a session key, which is crucial for encrypting subsequent data transactions, thereby enhancing the overall security of the system.

### 3.1. Initialization Stage of System

Entering secure parameter n chosen randomly and running the key generation algorithm $\left(pk,sk)\leftarrow KeyGen(1^{n}\right)$ who needs to obtain public–private key pairs. Thereafter, the SDN controller picks B0←Rr and carries on trapdoor generation algorithm additionally to acquire $(B_{1},T_{1})\leftarrow ideal-trapGen$ and $(B_{2},T_{2})\leftarrow ideal-trapGen$. Ultimately, acquisition of the public key $pk=(B_{0},B_{1},B_{2})$ and private key $sk=(T_{1},T_{2})$ occurs. Note that $pk=\{B_{0},B_{1},B_{2}\}$ overt. Additionally, the SDN controller establishes quantum key distribution (QKD) links with both the OLT and ONUs that uses the BB84 protocol to generate and share a secure quantum key. To ensure the security of the public key pk, the SDN controller shares the key with the ONU/OLT through the BB84 protocol to encrypt the pk. The encrypted public key is sent to the ONU/OLT, which uses the same shared key to decrypt it, ensuring that the correct public key pk is obtained. These QKD links will be used to enhance the security of subsequent authentication processes and provide confidentiality and integrity by detecting any potential eavesdropping.

### 3.2. Both OLT and ONU Register with SDN Controller (Take ONU as an Example)

**Figure 1:**
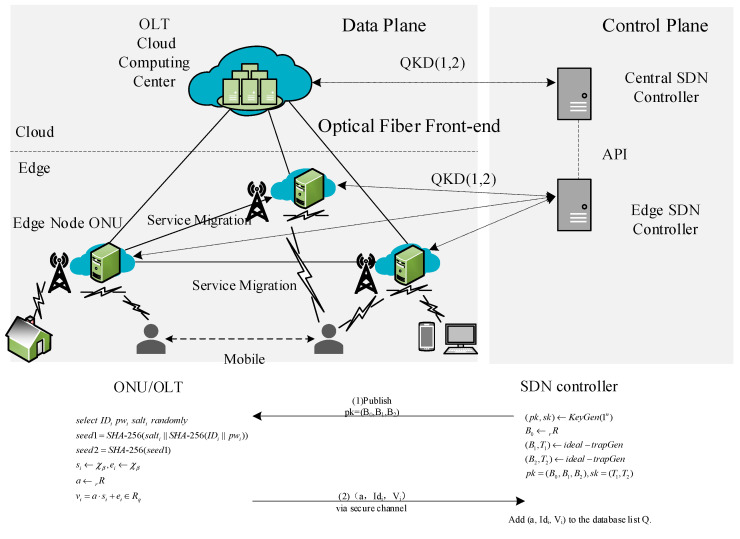
OLT and ONU registration with SDN controller.

ONU and OLT need to register with the SDN controller first when they perform key negotiation with the help of the SDN controller, as illustrated in Figure 1.

(1) ONU selects $ID_{i}$, $pw_{i}$, and $salt_{i}$ at random, and in the same breath, it adopts SHA-256 to produce seeds of two pseudo-random number generators. Seeds generated are as follows:
  (1) $$seed_{1}=SHA\text{-}256(salt_{i}∥SHA\text{-}256(ID_{i}∥pw_{i})),$$
  (2) $$seed_{2}=SHA\text{-}256(seed_{1}).$$
(2) Enter the seeds that were obtained by step 1, and thereafter choose polynomials $s_{i}\leftarrow \chi_{\beta },e_{i}\leftarrow \chi_{\beta }$ and select a←R at random. Compute the verification element for ONU,
  (3) $$v_{i}=a\cdot s_{i}+e_{i}\in R_{q}.$$

ONU sends $\left(a,ID_{i},v_{i}\right)$ to the SDN controller via a secure channel. It should be highlighted that the ONU/OLT, in the process of sending the registration message to the SDN controller, encrypts the verification element $v_{i}$ to obtain $v_{i}^{\prime }={\mathrm{Enc}}_{K_{share}}(v_{i})$ using the key $K_{share}$ that is securely and secretly shared with the SDN controller through the QKD protocol, and sends the encrypted registration message $\left(a_{i},{\mathrm{Enc}}_{K_{share}}(ID_{i}),v_{i}^{\prime }\right)$ to the SDN controller, which decrypts it to obtain the verification element $v_{i}={\mathrm{Dec}}_{K_{share}}(v_{i}^{\prime })$ to securely obtain the correct registration message $\left(a,ID_{i},v_{i}\right)$. The message will be instinctively written into the database list Q if it not included in Q. Or else SDN controller will ask the ONU to send a novel registration message again. After the ONU has been successfully registered, $\left(seed_{1},seed_{2},s_{i},e_{i},v_{i}\right)$ is deleted and $\left(pw_{i},salt_{i}\right)$ is saved locally. Eventually, the SDN controller has a password list $pw_{i}=\{salt_{i},v_{i}\}$ of all ONUs and OLT, where the verification element $v_{i}$ is composed of their salt value salti and the password $pw_{i}$.

### 3.3. Mutual Authentication in the ONUs Auto-Discovery Process

**Figure 2:**
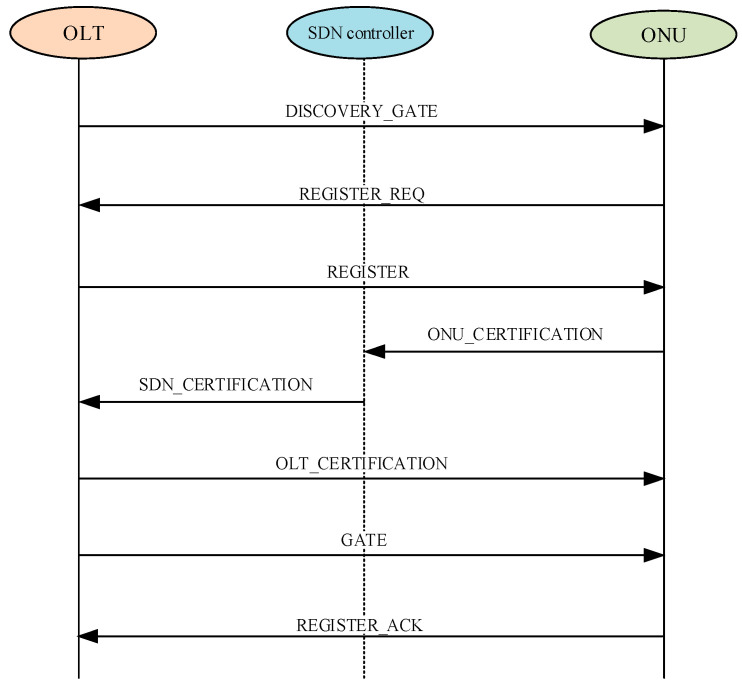
The process of authentication mechanism.

Following the registration of the optical line terminal (OLT) and optical network units (ONUs) with the software-defined networking (SDN) controller, the ONUs are required to register with the OLT upon joining the ethernet passive optical network (EPON) system. Throughout this process, both the OLT and ONUs engage in session key negotiation, facilitated by the SDN controller. The authentication mechanism of the EPON system is illustrated in Figure 2. The detailed steps for the implementation are as follows:

(1) The OLT broadcasts DISCOVERY_GATE frame to all ONUs in the system every one second with an all-zero LLID destination address, informing the ONUs of the permitted start time for transmission and the permissible length. The EPON network management controls whether the registration authorization frame is activated. When the OLT receives the activation information from network management, it starts to periodically send the registration authorization information. Similarly, the OLT stops sending the registration authorization frame after receiving the stop information.
(2) Upon receiving the DISCOVERY_GATE message from OLT, the ONU that applied for registration will return REGISTER_REQ frame to OLT within its corresponding discover slot allocated by authorization. If the ONU does not receive a response from the OLT after 100 ms of sending the registration request frame, it is assumed that a registration conflict has arisen and the registration request is re-sent after a delay period.
(3) After receiving the frame at the OLT side, not only does OLT assign an ONU_ID to the ONU but also sends REGISTER frame in broadcast mode with the destination MAC address pointing to it. It must be noted here that OLT will dispose of it if only one registration request frame is received in the same windowing period.

ONU selects $e_{1},e_{2},e_{3}$ from $R^{m},\;w_{ONU},sk\prime_{ONU}$ from $R_{q}$; in addition, ONU generates hash key $hk_{ONU}$ (4), verification element ${v^{\prime }}_{ONU}$ (5), and message $m_{ONU}$ (6). Thereafter, ONU performs an encryption operation on $m_{ONU}$ with pk to obtain the cipher text $C_{ONU1}$, $C_{ONU2}$ (7) and calculates the hash function value $h_{ONU}$ (8) on the basis of $pk,\;hk_{ONU},\;C_{ONU1},\;m_{ONU}$. In Equation (9), where the temporary private key $sk\prime_{ONU}$ and $\;x_{ONU}$ is applied to make $\varphi_{ONU}$ dynamic and distinctive in each varied discover time window, (10), that is ONU_CERTIFICATION, is sent to SDN controller by ONU.

(4) $$hk_{ONU}=HashKG(1^{n}),$$

(5) $${v^{\prime }}_{ONU}=a\cdot s_{ONU}+e_{ONU},$$

(6) $$m_{ONU}=(ID_{ONU}∥ID_{OLT}∥SDN∥hk_{ONU}∥ssid_{ONU}∥{v_{ONU}}^{\prime }∥1\ldots ),$$

(7) $$C_{ONU1}=B_{1}\cdot w_{ONU}+e_{ONU}(\mathrm{mod}q),\;C_{ONU2}=B_{0}\cdot w_{ONU}+B_{2}\cdot m_{ONU}+e_{2}(\mathrm{mod}q),$$

(8) $$h_{ONU}=Hash(pk,hk_{ONU},C_{ONU1},m_{ONU}),$$

(9) $$x_{ONU}=a\cdot s{k^{\prime }}_{ONU}+2e_{3}\in R_{q},\varphi_{ONU}=MAC(h_{ONU},ID_{ONU}∥ID_{OLT}∥SDN∥x_{ONU}),$$

(10) $$<ID_{ONU},ID_{OLT},SDN,hk_{ONU},C_{ONU}=(C_{ONU1},C_{ONU2}),ssid_{ONU},x_{ONU},\varphi_{ONU}>,$$

(4) When the SDN controller receives the message (10) from ONU, ONU first searches for the existence of the ONU corresponding to *v*_*ONU*_ in the local list pws then checks if the session serial number $ssid_{ONU}$ meet the requirements. If both meet the requirements, the next SDN controller recovers $m_{ONU}$ and $h_{ONU}$ according to the message that is sent by ONU. The SDN controller restores the $m_{ONU}$ by the ${v^{\prime }}_{ONU}$ of the local list and verifies the range of ciphertext ($C_{ONU1},C_{ONU2}$) to realize the identity authentication of ONU.
(5) The SDN controller $\delta_{sONU},\delta_{sOLT}\leftarrow \left\{0,1\right\},e_{4},e_{5},w_{OLT}\leftarrow R$, where $e_{4},e_{5}$ are polynomials whose coefficients follow discrete distribution $\chi_{\beta }$. The hash key $hk_{OLT}$ (11) is selected by OLT randomly; furthermore, the SDN controller calculates projection key $hp_{OLT}$ (12), projection function value $h_{OLT}$ (13), and message (14) and encrypts $m_{OLT}$ with pk to obtain the ciphertext (15). Afterwards, the SDN controller computes Equations (16) and (17) by random values $\delta_{sONU},\delta_{sOLT}$. Ultimately, the SDN controller uses projection function value $h_{OLT}$ generate evidential MAC value (18) and sends message (19), i.e., SDN_CERTIFICATION to OLT.
  (11) $$hk_{OLT}=HashKG(1^{n}),$$
  (12) $$hp_{OLT}=\mathrm{Pr}ojKG(pk,hk_{OLT}),$$
  (13) $$h_{OLT}=\mathrm{Pr}ojH(hp_{OLT},w_{OLT}),$$
  (14) $$m_{OLT}=(ID_{OLT}∥ID_{\mathrm{ONU}}∥SDN∥hk_{OLT}∥ssid_{OLT}∥v_{OLT}∥1\ldots ),$$
  (15) $$C_{OLT1}=B_{1}\cdot w_{OLT}+e_{4}(\mathrm{mod}q),\;C_{OLT2}=B_{0}\cdot w_{OLT}+B_{2}\cdot m_{OLT}+e_{5}(\mathrm{mod}q),$$
  (16) $$c_{ONU}=F_{\delta_{sOLT}}(1)⊕F_{\delta_{sONU}}(3),c_{OLT}=F_{\delta_{sONU}}(1)⊕F_{\delta_{sOLT}}(3),$$
  (17) $$\Delta_{ONU}=h_{ONU}⊕ECC(\delta_{sONU}),\Delta_{OLT}=h_{OLT}⊕ECC(\delta_{sOLT}),$$
  (18) $${\varphi_{ONU}}^{\prime }=MAC(h_{OLT},ID_{ONU}∥ID_{OLT}∥SDN∥x_{ONU}),$$
  (19) $$<(c_{ONU},\Delta_{ONU},h_{ONU}),(ID_{ONU},ID_{OLT},SDN),c_{OLT},\Delta_{OLT},hk_{SDN},C_{OLT}=(C_{OLT1},C_{OLT2}),x_{ONU},{\varphi_{ONU}}^{\prime }>.$$
(6) OLT will recover verification element (20), message (21), and hash function value (22) as soon as receives the message sent from SDN controller. OLT can verify if ${\varphi_{ONU}}^{\prime }$ is correct through ${h_{OLT}}^{\prime }$ according to the correctness of the approximately smooth projection hash function.
  (20) $${v_{OLT}}^{\prime }=a\cdot s_{OLT}+e_{OLT},$$
  (21) $${m_{OLT}}^{\prime }=(ID_{OLT}∥ID_{ONU}∥SDN∥hk_{OLT}∥ssid_{OLT}∥{v_{OLT}}^{\prime }∥1\ldots ),$$
  (22) $${h_{OLT}}^{\prime }=Hash(pk,hk_{OLT},C_{OLT1},{m_{OLT}}^{\prime }).$$
(7) After verification is finished, OLT chooses $e_{6},e_{7},s{k_{OLT}}^{\prime }\leftarrow R$ randomly, where the coefficients of $e_{6},e_{7}$ follow Gaussian distribution and $s{k_{OLT}}^{\prime }$ is considered as a temporary private key of OLT. OLT calculates (23)–(24), computes (25), and generates the session key (26) with ONU and the verifiable MAC value (27) on the basis of ${h_{OLT}}^{\prime }$, the decoding algorithm ${\mathrm{ECC}}^{-1}$ of the error correction code and $\Delta_{OLT}$. OLT sends OLT_CERTIFICATION frame, Formula (28), to ONU at last. And OLT sends GATE frame to ONU in order to let ONU return the REGISTER_ACK frame.
  (23) $$x_{OLT}=a\cdot s{k_{OLT}}^{\prime }+2e_{6},k_{OLT}=x_{1}\cdot s{k_{OLT}}^{\prime }+2e_{7},$$
  (24) $$\sigma_{OLT}=g(k_{OLT}),\rho_{OLT}=Extr(k_{OLT},\sigma_{OLT}),$$
  (25) $$\delta_{SOLT}=ECC^{-1}({h_{OLT}}^{\prime }⊕\Delta_{OLT}),$$
  (26) $$SK_{OLTONU}=c_{OLT}⊕F_{\delta_{SOLT}}(1)⊕F_{\delta_{SOLT}}(3)⊕\sigma_{OLT}⊕\rho_{OLT},$$
  (27) $$\varphi_{OLT}=MAC(h_{OLT},ID_{ONU}∥ID_{OLT}∥SDN∥x_{OLT}),$$
  (28) $$<(c_{ONU},\Delta_{ONU},x_{OLT}),(ID_{ONU},ID_{OLT},SDN),\sigma_{OLT},\varphi_{OLT},ssid_{OLT}>.$$
(8) First of all, ONU checks the session ID $ssid_{OLT}$. In addition, ONU will compute the corresponding projection key $hp_{ONU}$ (29) according to $hp_{ONU}$ that is stocked locally if $ssid_{OLT}$ meets the requirement. What is more, ONU calculates projection function value (30) in accordance with $hp_{ONU}$ and the evidence $w_{ONU}$, which can prove the ciphertext. ONU can verify if $\varphi_{OLT}$ is correct through ${h_{ONU}}^{\prime }$ according to the correctness of the approximately smooth projection hash function. ONU selects e8 irregularly and calculates (31) on the basis of ${h_{ONU}}^{\prime }$, the decoding algorithm ${\mathrm{ECC}}^{-1}$ of the error correction code and $\Delta_{ONU}$ after going through authentication. At last, ONU generates the session key (32) with OLT and transmits REGISTER_ACK frame to the OLT.
  (29) $$hp_{ONU}=\mathrm{Pr}ojKG(pk,hk_{ONU}),$$
  (30) $${h_{ONU}}^{\prime }=\mathrm{Pr}ojH(hp_{ONU},w_{ONU}),$$
  (31) $$\delta_{sONU}=ECC^{-1}({h_{ONU}}^{\prime }⊕\Delta_{ONU}),k_{ONU}=y\cdot s{k_{ONU}}^{\prime }+2e_{8},\rho_{ONU}=Extr(k_{ONU},\sigma_{OLT}),$$
  (32) $$SK_{ONUOLT}=c_{ONU}⊕F_{\delta_{sONU}}(1)⊕F_{\delta s_{ONU}}(3)⊕\sigma_{OLT}⊕\rho_{ONU}.$$
(9) If the REGISTER_ACK frame is received from the ONU after a period of time has elapsed since the GATE frame was sent, then the ONU is considered to be successfully registered. Upon successful registration, the ONU and the OLT can share the session key (33) for the following interaction.
  (33) $$SK_{ONUOLT}=F_{\delta_{sOLT}}(1)⊕F_{\delta_{sONU}}(1)⊕\sigma_{OLT}⊕\rho_{OLT}=F_{\delta_{sOLT}}(1)⊕F_{\delta_{sONU}}(1)⊕\sigma_{OLT}⊕\rho_{ONU}.$$

So far, the automatic discovery and registration process based on authentication has been completed. The flowchart of the whole authentication process is depicted in Table 1.

## 4. Security Performance Evaluation

In this section, we compare the performance of our strategy with other similar schemes in terms of security and efficiency.

### 4.1. Strength Against Attacks

Table 2 provides comparison results of security with references [10,13,26,27,33], where the difficulty problem indicates the difficult issue on which the agreement is based, type represents the protocol type, and the rest denote attacks that the protocol can resist. Our scheme offers significant advantages over other schemes [10,13,26,27,33]. First, it supports a three-party protocol, providing greater flexibility, while most other schemes support only two-party protocols. Second, our scheme effectively prevents relay attacks, known key secrecy attacks, and provides forward security, features that are not protected in [10,13,26,27,33]. Most importantly, the encryption mechanism based on the RLWE problem provides strong resistance to quantum computing attacks, ensuring future security, while other schemes [10,26,27,33] face potential risks from quantum computing. Therefore, our scheme offers more comprehensive protection in various attack scenarios and provides long-term security assurances.

(1) **For man-in-the-middle attack:** The man-in-the-middle attack in this paper refers to an attacker in an EPON network that takes advantage of the inability of the OLT, ONU, and SDN controller to determine each other’s identity. In this scenario, if an attacker wants to launch a man-in-the-middle attack, it must first intercept the messages transmitted by them, but the attacker cannot get $h_{i}$ through $\varphi_{i}$, and thus cannot learn the message $m_{i}$, nor the authentication element $v_{i}$ that can verify identity. On one hand, the attacker in the quantum network intercepts the communication between the SDN controller and ONU/OLT. Through QKD protocol, the symmetric keys $K_{share}$ are securely distributed between the parties. Any attempt to intercept the key exchange will be immediately detected due to the quantum nature of the communication, which causes the quantum states to collapse and reveal the presence of an eavesdropper. This prevents an attacker from impersonating the SDN, ONU/OLT, ensuring the authenticity of the key exchange. Therefore, QKD’s inherent detection of eavesdropping makes it highly effective in defending against eavesdropping and man-in-the-middle attacks. On the other hand, and even if $v_{i}$ is compromised, the attacker cannot successfully execute the attack on account of the fact that the private key $s_{i}$ is confidential. As a result, it is difficult for the attacker to conduct a man-in-the-middle attack in bounded time.

(2) **For impersonation attack:** Impersonation attack refers to an illegal user masquerading as a legitimate ONU to be registered for authentication with the OLT or an illegal user masquerading as a legitimate OLT or SDN controller to change system information. In this scheme, the system is able to complete the authentication of the other communication entity before the end of the ONU registration process. If the authentication fails, the illegal user will be detected, then the registration is terminated and the attacker is unable to carry out subsequent operations. Therefore, the protocol can resist the masquerade attack effectively.

(3) **For replay attack:** It means that the attacker sends a packet that has been received by the destination host to cheat the system. In this scheme, a new session sequence number $ssid_{i}$ will be automatically generated for each session initiated by the ONU and OLT. After each session is successfully executed, the SDN controller will record it in the local list to prevent message replay. In addition, the temporary private keys $sk_{i}$ will be randomly selected every time authentication is performed, and these temporary private keys are invisible to the attacker. Accordingly, the generated authentication parameters $x_{i}$, $\varphi_{i}$ are random every time, so that an attacker cannot perform a replay attack based on previously sent legitimate authentication messages.

(4) **Known key secrecy attack:** It means that when an attacker obtains or cracks an expired session key (32), he can break the new session key again within a limited time. In this scheme, OLT and ONU will generate a new session key at the end of each authentication, in which $\delta_{sONU},\delta_{sOLT}$ is randomly selected by the SDN controller, $k_{ONU}$ and $k_{OLT}$ are related to the temporary private key $sk_{i}^{\prime }$ of ONU and OLT, respectively, and $sk_{i}^{\prime }$ that is not transmitted on the channel is random and confidential, so the session key changes constantly and the known session key attack is invalid.

(5) **Forward security:** It means that even if an attacker somehow obtains the long-term private key for each participant, it is still impossible to calculate the previous key that the OLT and ONU successfully negotiated. In this scenario, the session key for the ONU and the OLT needs to be generated with the help of the SDN controller. The complete session key consists of three parts: one for the ONU, one for the OLT, and the other is determined by the SDN controller. When an attacker has the long-term private key of the SDN controller, it can authenticate to the ONU, the OLT and the SDN controller by forging ciphertexts and signatures. But they can only have access to this part of the session key calculated by the SDN controller, and the session keys for both the ONU and the OLT are also associated with the temporary key they choose. Whereas the temporary private key is not transmitted over the channel, an attacker can only obtain A that is transmitted over the channel, so the scheme has forward security.

### 4.2. Influence on Registration Efficiency

The standard automatic discovery and registration process for Optical Network Units (ONUs) does not encompass identity authentication. Incorporating an authentication algorithm between ONUs and Optical Line Terminals (OLTs) is anticipated to augment transmission and processing delays, potentially reducing the number of successful ONU registrations. To assess the performance implications of this authentication algorithm more intuitively, we conducted simulation experiments.

**Simulation Setup:** These experiments were executed in a Windows 10 environment, utilizing an AMD Ryzen 7 5800U @1.90 GHz processor and 16.0 GB of RAM, employing Java as the programming language. The simulation framework was built to emulate a standard EPON network with a point-to-multipoint topology. The network includes one OLT and up to 100 ONUs, coordinated by an SDN controller for message exchange. Evaluation metrics: (1) System Delay: Measures the time delay introduced by the authentication scheme in the registration process. The delay is calculated as the difference between the timestamp of the first message sent by the ONU and the timestamp of the last acknowledgment received from the OLT. (2) Registration Success Rate: Defined as the ratio of successfully registered ONUs to the total number of registration attempts within a specific time window (Table 3).

**Test cases and experimental scenarios:** In order to test the scalability and robustness of the proposed scheme, three load scenarios are designed: a low load scenario with 10 ONUs attempting to register, a medium load scenario with 35 ONUs registering at the same time, and a high load scenario that simulates 60 ONUs initiating the registration process at the same time in order to test the system’s performance under high load. In addition, complex network conditions including message loss rates of 1%, 5%, and 10%, and artificial network delays of 10 ms, 50 ms, and 100 ms are introduced in each load scenario to simulate real network environments and evaluate the performance and robustness of the scheme under different load and anomaly conditions.

This study evaluated the effects of the authentication algorithm on system latency, execution stability, and the registration success rate across the three schemes under varying load conditions.

**Figure 3:**
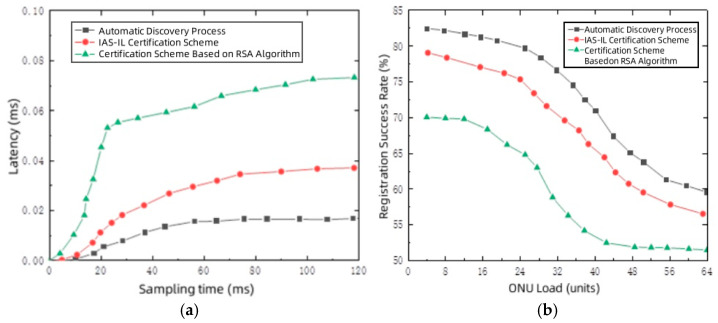
The subplot (**a**) illustrates the delay comparison of different algorithms, and the subplot (**b**) demonstrates the comparison of registration success rate of different algorithms.

Figure 3a presents a comparative analysis of delays associated with the IAS-IL authentication scheme, the RSA-based authentication scheme, and the standard automatic discovery process. The figure illustrates that system delay escalates with increasing simulation time, eventually reaching a stabilization point. The RSA signature authentication algorithm, due to its substantial computational demands, incurs a more significant delay. Despite the incorporation of a third party in our scheme, identity authentication is accomplished with minimal communication rounds with the SDN controller, keeping the delay consistently within 0.04 ms. This level of delay is negligible compared to the delay of the original registration process. In contrast, the delay of the RSA-based scheme rises significantly with the increase in load. This trend is mainly due to the advantages of our proposed scheme architecture. By introducing the SDN controller as a centralized processing unit, computationally intensive tasks such as authentication and key negotiation are offloaded to the SDN controller, which significantly reduces the computational burden on the ONUs and ensures low latency and high scalability. In contrast, traditional RSA-based schemes rely on local computation per ONU, and as the number of ONUs in the system increases, performance bottlenecks become more pronounced, resulting in higher delay. Although the implementation of the authentication algorithm marginally increases system delay, it substantially enhances the system’s reliability by ensuring the verification of the legitimacy of ONUs and OLTs. These results show that the proposed scheme is able to maintain stable performance under high load conditions and is particularly suitable for large-scale EPON deployment environments.

Figure 3b illustrates the registration success rates for the IAS-IL authentication scheme, the RSA-based authentication algorithm, and the automatic discovery process. It can be observed from the graph that as the load continuously increases, which means as the number of ONUs requiring registration within the system grows, the likelihood of registration frame collisions also increases. Consequently, regardless of the method used, the registration success rate of ONUs will decrease under high load conditions. However, the success rate of this scheme is significantly better than that of the RSA-based authentication scheme, remaining above 55%, which is not much different from the automatic discovery process. Our scheme demonstrates higher throughput under heavy load, thanks to the SDN controller’s centralized task processing architecture. In contrast, the traditional scheme experiences a decline in throughput as the load increases, due to local computation bottlenecks. This trend indicates that our proposed scheme performs better in terms of throughput in large-scale networks.

## 5. Conclusions

In this paper, we have presented an ethernet passive optical network (EPON) mutual authentication scheme based on ideal lattices. The scheme utilizes the security of the ring learning with errors (RLWE) problem to ensure the robustness of the public-key cryptosystem. By incorporating the approximate smooth projection hash function, the proposed scheme enables secure key exchange and mutual authentication between ONUs and OLTs with the assistance of an SDN controller. The evaluation of the scheme’s security performance against various attacks, including man-in-the-middle, impersonation, replay, and known key secrecy attacks, demonstrates its resilience and effectiveness.

Furthermore, the simulation results show that the proposed authentication scheme introduces minimal delay and maintains a high registration success rate, even under high load conditions. This is attributed to the efficient utilization of the approximate smooth projection hash function, which allows for secure authentication with only two communications with the SDN controller. Overall, the proposed EPON mutual authentication scheme based on ideal lattices provides a secure and efficient solution for identity authentication in EPON networks.

Moreover, Quantum Key Distribution (QKD) introduces robust security into EPON networks, but its implementation faces several challenges due to hardware limitations. QKD relies on single-photon sources, single-photon detectors (SPDs), and low-loss quantum communication channels. Current SPDs, such as avalanche photodiodes, have limited efficiency and high dark count rates, which constrain the key generation rate. Additionally, hybrid communication channels must transmit quantum and classical signals over the same fiber, requiring precise synchronization and advanced multiplexing techniques to minimize cross-talk. The hardware requirements for QKD include high-efficiency SPDs, stable single-photon sources, and quantum signal multiplexers/demultiplexers. Time synchronization units are essential to ensure accurate photon detection. Despite these advancements, challenges such as optical loss in long-distance communication and high initial costs of quantum hardware persist, impacting system scalability and performance. Future developments, such as integrated quantum photonic chips, may alleviate these challenges and facilitate large-scale deployment.

Moving forward, we plan to further explore the integration of QKD technology into EPON systems and address the remaining challenges in quantum communication hardware. We aim to improve the scalability and cost efficiency of our scheme while maintaining its robust security guarantees. This research provides a promising foundation for developing secure, quantum-resistant communication protocols for next-generation optical networks.

## Figures and Tables

**Table 1:** The whole authentication scheme.

***Scheme***
***Initial Stage***
$\mathrm{SDN}\;\mathrm{sample}\;B_{0}\leftarrow trapGen$, *acquire* $(B_{1},T_{1}),(B_{2},T_{2})\leftarrow ideal-trapGen$, *set* $pk=(B_{0},B_{1},B_{2})$, $sk=(T_{1},T_{2})$, *send* pk to ONU.
***Register Stage***
*1.* $\mathrm{ONU}\;\mathrm{select}\;ID_{i},pw_{i},salt_{i}$, *generate* $seed_{1},seed_{2}$.
*2. Sample* $s_{i}\leftarrow \chi_{\beta },e_{i}\leftarrow \chi_{\beta },a\leftarrow R$ *and set* $v_{i}=a\cdot s_{i}+e_{i}$
*3. Sends* $\left(a,ID_{i},v_{i}\right)$ *to* SDN.
***Authentication Stage***
*1.* OLT *broadcasts* DISCOVERY_GATE *frame*.
*2. If* ONU *has registered*, ONU *returns* REGISTER_REQ *frame*.
*3.* OLT *assign* ONU_ID *to* ONU *and broadcasts* REGISTER *frame*. $\mathrm{ONU}\;\mathrm{runs}\;Enc(pk,m_{ONU})=C_{ONU1},C_{ONU2}$ *and* $ASPH(pk,hk_{ONU},C_{ONU1},m_{ONU})$, *computes* $x_{ONU}$ *and* $\varphi_{ONU}$. ONU *sends* ONU_CERTIFICATION *to* SDN.
*4.* SDN *checks* $v_{ONU}$ *and* $ssid_{ONU}$, *recovers* $m_{ONU},h_{ONU}$, *restores* $m_{ONU}$ *by* ${v^{\prime }}_{ONU}$, *and realize identity authentication in ciphertext* $C_{ONU}$.
*5.* SDN *runs* $hk_{OLT}=HashKG(1^{n})$, $hp_{OLT}=\mathrm{Pr}ojKG(pk,hk_{OLT})$, $h_{OLT}=\mathrm{Pr}ojH(hp_{OLT},w_{OLT})$$\;\mathrm{and}\;\mathrm{computes}\;m_{OLT},C_{OLT1},C_{OLT2},c_{ONU},c_{OLT},\Delta_{ONU},\Delta_{OLT}$. SDN *send* SDN_CERTIFICATION *frame* *to* OLT.
*6.* $\mathrm{OLT}\;\mathrm{recovers}\;{v^{\prime }}_{OLT},{m^{\prime }}_{OLT},{h^{\prime }}_{OLT}$ *when receive the message and begins the Verification*.
*7.* OLT *computes* $x_{OLT},k_{OLT},\sigma_{OLT},\delta_{SOLT},SK_{OLTONU}$ *and the verifiable value* $\varphi_{OLT}$. OLT *sends* OLT_CERTIFICATION *frame* *and* GATE *frame to* ONU.
*8.* $\mathrm{ONU}\;\mathrm{checks}\;ssid_{OLT}$, *computes* $hp_{ONU},{h^{\prime }}_{ONU},\delta_{sONU}$ *and generates* $SK_{ONUOLT}$ *with* OLT. ONU *sends* REGISTER_ACK *frame to* OLT.
*9.*$\;\mathrm{Upon}\;\mathrm{successful}\;\mathrm{registration},\;\mathrm{ONU}\;\mathrm{and}\;\mathrm{OLT}\;\mathrm{can}\;\mathrm{share}\;\mathrm{the}\;\mathrm{session}\;\mathrm{key}\;\mathrm{by}\;SK_{ONUOLT}=F_{\delta_{sOLT}}(1)⊕F_{\delta_{sONU}}(1)⊕\sigma_{OLT}⊕\rho_{OLT}=F_{\delta_{sOLT}}(1)⊕F_{\delta_{sONU}}(1)⊕\sigma_{OLT}⊕\rho_{OLT}$.

**Table 2:** Security comparison.

Scheme	[10]	[26]	[27]	[33]	[13]	Ours
Difficulty problem	GCM	ECC	RSA	(ECC/RSA) + AES	NTRU	RLWE
Type	3-party	2-party	2-party	2-party	2-party	3-party
Man-in-the-middle attack	√	√	√	√	√	√
Impersonation attack	√	√	√	√	√	√
Relay attack	×	×	×	×	√	√
Known key secrecy attack	×	×	×	×	×	√
Forward security	×	×	×	×	×	√

**Table 3:** Software and hardware environment for experiments.

Type	Environment
Operating System	Windows10
CPU	AMD Ryzen 7 5800U @1.90 GHz
Memory	16 GB
Development Tool	IDEA 2024
Development Language	Java

## Data Availability

The original contributions presented in this study are included in the article; further inquiries can be directed to the corresponding author.

## Abbreviations

**Notation**	**Definition**
$ID_{i}$	Identification of i
$pw_{i}$	password of i
$salt_{i}$	Salt value of i
$v_{i}$	Verification element of i
$SK_{ik}$	Session key of *i* and *k*
$hk_{i}$	Hash key of i
$hp_{i}$	Projection key of i
$h_{i}$	Hash function value of i
$h_{i}^{\prime }$	Projection function value of i
$pk_{i}$	Public key of i
$sk_{i}$	Private key of i
$\varphi_{i}$	Verifiable MAC value of i
$\chi_{\beta }$	Discrete Gaussian distribution
$m_{i}$	The plaintext message sent by the communicating entity i
$C_{i}$	The ciphertext message sent by the communicating entity i
$ssid_{i}$	Session id of i
$R_{q}$	Polynomial rings with integral coefficients modulo q
i||k	i and k are connected bin series
